# The effect of phytochemicals in N-methyl-N-nitro-N-nitroguanidine promoting the occurrence and development of gastric cancer

**DOI:** 10.3389/fphar.2023.1203265

**Published:** 2023-06-29

**Authors:** Ling Lu, Bei Chen, XinYi Zhang, Yumeng Xu, Longtao Jin, Hui Qian, Zhao feng Liang

**Affiliations:** ^1^ Child Healthcare Department, The Fourth Affiliated Hospital of Jiangsu University, Zhenjiang, Jiangsu, China; ^2^ Jiangsu Key Laboratory of Medical Science and Laboratory Medicine, School of Medicine, Jiangsu University, Zhenjiang, Jiangsu, China

**Keywords:** gastric cancer, phytochemicals, prevention, MNNG, mechanisms

## Abstract

Gastric cancer is a common malignant tumor of the digestive tract, with a low early diagnosis rate. N-methyl-N-nitro-N-nitroguanidine (MNNG) is one of the main risk factors for gastric cancer. Phytochemicals are healthy active substances derived from vegetables, fruits, nuts, tea, herbal medicines and other plants. Taking phytochemicals is a very promising strategy for the prevention and treatment of gastric cancer. Many studies have proved that phytochemicals have protective effects on MNNG induced gastric cancer via inhibiting cell proliferation, enhancing immunity, suppressing cell invasion and migration, inducing apoptosis and autophagy, blocking angiogenesis, inhibiting *Helicobacter pylori* infection as well as regulating metabolism and microbiota. The intervention and therapeutic effects of phytochemicals in MNNG induced gastric cancer have attracted more and more attention. In order to better study and explore the role, advantages and challenges of phytochemicals in MNNG induced gastric cancer, we summarized the intervention and therapeutic effects of phytochemicals in MNNG induced gastric cancer. This review may help to further promote the research and clinical application of phytochemicals in MNNG induced gastric cancer, and provide some new insights.

## 1 Introduction

Gastric cancer is one of the most common malignant tumors worldwide, which is a multicentric pathological process caused by multiple risk factors (Sung et al., 2021). N-nitrosamines is one of the main risk factors of gastric cancer, which widely exists in the living environment and various food. N-methyl-N-nitro-N-nitroguanidine (MNNG) is a common N-nitrosamine chemical that is often used to study the role and mechanism of N-nitrosamines in inducing gastric cancer (Cai et al., 2019; Liang et al., 2022a). Studies have showed that MNNG promotes the occurrence of gastric cancer (Isyraqiah et al., 2019; Lin et al., 2019; Gunes-Bayir et al., 2022). The lack of effective diagnostic markers and treatment methods leads to the fact that patients with gastric cancer are often in the advanced stage when diagnosed and have a poor prognosis. Therefore, there is an urgent need to explore the less toxic and more effective strategies or methods for preventing and treating gastric cancer, especially MNNG-induced gastric cancer.

**FIGURE 1:**
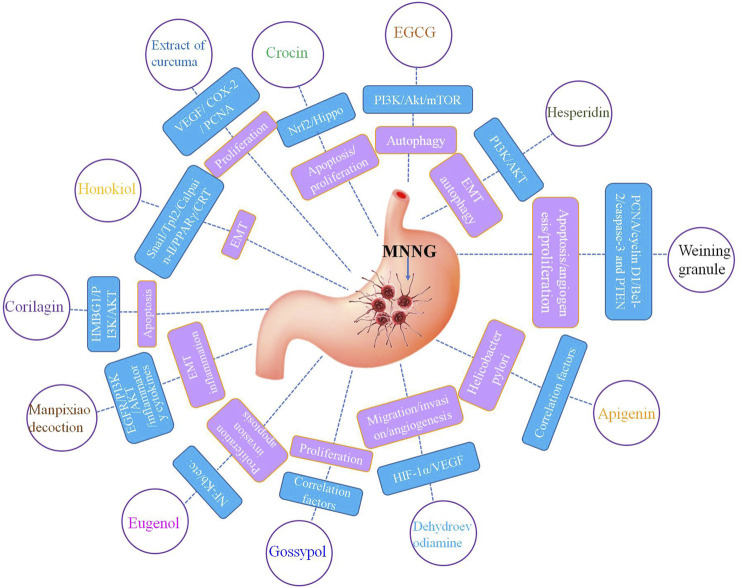
Phytochemicals have protective effects against MNNG induced gastric cancer.

As a natural product, phytochemicals have a variety of biological activities and excellent health effects in a lot of physiological and pathological processes. Vegetables, fruits, tea, spices, nuts, soybeans, edible fungi and grains are rich in phytochemicals (Bastos et al., 2010; Mao et al., 2020; Liang et al., 2022b). Supplement of phytochemicals have been proved to be a safe and promising method for the prevention and treatment of cancers such as gastric cancer (Lu et al., 2016; Liang et al., 2022a). Studies have demonstrated that phytochemicals have excellent efficacy in the prevention and treatment of MNNG related gastric cancer (Nagata et al., 2002; Bastos et al., 2010; Mao et al., 2020) (Figure 1). Phytochemicals have protective and therapeutic effects against MNNG induced gastric cancer through inhibiting cell proliferation, suppressing cell invasion and migration, anti-angiogenesis, inducing cell apoptosis and autophagy, affecting inflammation, oxidative stress and immunity, regulating metabolism and flora, and other mechanisms.

This article reviewed the intervention and treatment of phytochemicals on MNNG-induced gastric cancer, and also discussed the underlying mechanisms. At the same time, the shortcomings, challenges and future research directions of phytochemistry were expounded in the intervention and treatment of gastric cancer.

## 2 Effects of N-nitrosamines and MNNG on the occurrence and progression of gastric cancer

N-nitrosamines are closely related to the occurrence and development of various cancers, especially gastrointestinal tumors such as gastric cancer (Taneja et al., 2017; Hidajat et al., 2019). Studies have indicated that long-term low-dose exposure to N-nitrosamine is one of the main causes of gastric cancer, which promotes gastric cancer occurrence and development (Vermeer et al., 2001; Vermeer et al., 2002). Researchers used MNNG to simulate N-nitrosamines to explore the effect and mechanism of MNNG in the occurrence and progression of gastric cancer (Furihata, 2021; Gunes-Bayir et al., 2022). MNNG can induce gastric cancer by regulating inflammation, cell damage, cell differentiation mitochondrial dysfunction, glycolysis pathogenesis and oxidative stress processes (Cai et al., 2018; Zhao et al., 2019a; Cai et al., 2019; Cui et al., 2021; Wen et al., 2021; Gunes-Bayir et al., 2022). MNNG promotes the occurrence and development of gastric cancer by regulating processes such as autophagy, EMT, apoptosis, proliferation, angiogenesis, gastric epithelial dysplasia, malignant transformation and change endogenous metabolites (Zhao et al., 2019b; Deng et al., 2019; Wu and Hui, 2020; Liang et al., 2022a; Cai et al., 2022; Chen et al., 2022; Peng et al., 2022; Yang et al., 2022; Xie et al., 2023).Therefore, there is an urgent need to find an effective and low side effect intervention or treatment strategy. In recent years, phytochemicals and active ingredients of herbal medicine have showed excellent application prospects in the prevention and treatment of gastric cancer.

## 3 Interventional effect of phytochemicals on MNNG induced gastric cancer

A large number of studies have showed that phytochemicals play a very important intervention effect on gastric cancer (Mao et al., 2020; Liang et al., 2022a; Zhao et al., 2023). The effects of phytochemicals on the treatment and prevention of MNNG exposure related gastric cancer also have been studied. MNNG induced gastric cancer is a typical representative of gastric cancer caused by dietary factors. Therefore, phytochemistry may play a better role in intervention and treatment of MNNG related gastric cancer.

### 3.1 Inhibition of uncontrolled cell proliferation induced by MNNG

Uncontrolled cell proliferation is one of the key steps in the MNNG-induced gastric cancer. A large number of phytochemicals have been shown to suppress the cell proliferation during the process of MNNG induced gastric cancer occurrence and development (Table 1).

**TABLE1:** Overview of the effect of phytochemicals on the proliferation of MNNG induced gastric cancer.

Phytochemicals	Effects	Target	Subjects	References
Weining granule	Inhibited cell proliferation	PCNA/cyclin D1	Rats	Deng et al. (2019)
Crocin	Inhibited cell proliferation	Nrf2/Hippo	Rats	Wu and Hui (2020)
Crocetin	Inhibited cell proliferation	Bcl-2/Bax	Gastric cancer cells and Rats	Bathaie et al. (2013a)
Extract of curcumae	Inhibited cell proliferation	VEGF, COX-2 and PCNA	Rats	Lu et al. (2010)
Gossypol	Inhibited cell proliferation	—	Gastric cancer cells and Rats	Gunassekaran et al. (2011)
Ginsenoside Rg3	Inhibited cell proliferation	TIGAR, GSH, NADP and G6PD	Gastric cancer cells	Lv et al. (2022)
Saffron aqueous extract	Inhibited cell proliferation	—	Rats	Bathaie et al. (2013b)
Eugenol	Inhibited cell proliferation	NF-κB	Rats	Manikandan et al. (2011)
Actinidia valvata Dunn extract	Inhibited cell proliferation	Bcl-2, cyclinD1, Bax and Caspase-3	Rats	Wang et al. (2014a)

Weining granule is a commonly used herbal medicine for treating stomach diseases. It was found that Weining granule improved gastric cancer by suppressing cell proliferation through regulating the expression of PCNA and cyclin D1 (Deng et al., 2019). Crocin is an active constituent of saffron, which has anticancer activity. It was reported that Crocin inhibited the proliferation of gastric cancer cells and blocked the cycle arrest induced by MNNG via Nrf2/Hippo (Wu and Hui, 2020). Crocetin is a carotenoid derived from saffron, which can inhibit MNNG induced abnormal proliferation of gastric cancer cells (Bathaie et al., 2013a). Curcuma longa extract could suppress the proliferation of gastric mucosal cells in the process of MNNG carcinogenesis and reduce the incidence rate of cancer by regulating the expression of PCNA (Lu et al., 2010). Gossypol is a highly effective polyphenol compound that has been proven to inhibit the proliferation of several cancer cells. Gunassekaran et al. (2011) found that Gossypol inhibited MNNG induced gastric cancer cell proliferation and the incidence of gastric cancer in animals. Ginsenoside Rg3 is one of the most effective active ingredients in ginseng extract, with a variety of biological effects. Lv et al. (2022) reported that Ginsenoside Rg3 could inhibit cell proliferation in MNNG induced gastric precancerous lesion rats. They found that after GRg3 treatment, the expression of TIGAR and the production of NADP, GSH G6PDH decreased, leading to the increase of ROS in the gastric mucosal epithelium, thereby inhibiting abnormal proliferation of gastric mucosal epithelial cells. Saffron water extract inhibits MNNG induced gastric cancer in Wistar rats by suppressing cell proliferation (Bathaie et al., 2013b). Eugenol is a natural phenolic component in clove oil, which has attractive therapeutic effects on a variety of tumors. In the MNNG induced gastric cancer rat model, Eugenol intervenes in the occurrence of gastric cancer by inhibiting NF-κB pathway and inhibiting cell proliferation (Manikandan et al., 2011). Actinidia valvata Dunn extract has a significant preventive effect on MNNG induced gastrointestinal cancer by regulating cell proliferation via regulating the expression of cyclin D1 (Wang et al., 2014a).

Abnormal cell proliferation has been showed to play a key role in the MNNG-triggered gastric carcinogenesis. Many phytochemicals or herbal medicines exhibit excellent anti-tumor effects at multiple stages of MNNG mediated gastric cancer by suppressing cell proliferation. The results of these above studies indicated that phytochemicals could be used as an application strategy for the MNNG related gastric cancer.

### 3.2 Suppression of the migration and invasion of cells elicited by MNNG

The migration and invasion of cells play critical effects in the initiation and development of MNNG-elicited gastric cancer. Enhanced migration and invasion ability predict poor treatment efficacy and patient prognosis (Guo et al., 2021). EMT plays a crucial role in the metastasis and invasion of gastric cancer by enhancing cell motility and invasiveness. Numerous signaling pathways, transcription factors, protein molecules, and genes have been reported to regulate cell migration, invasion, and EMT processes, thereby playing important roles in tumors such as gastric cancer. Phytochemicals may interfere with these signal pathways, transcription factors, protein molecules and genes in the process of gastric carcinogenesis and development.

More and more evidences have revealed that numerous phytochemicals inhibit cell migration and invasion induced by MMNG, thereby interfering with the initiation and progression of gastric cancer. As a quinazoline alkaloid isolated from Euodiae Fructus, Dehydroevodiamine inhibited MNNG induced migration and invasion of cells, interfering with the occurrence of gastric cancer by suppressing the HIF-1α/VEGF pathway (Wen et al., 2021). Dendrobium huoshanense is an important edible and medicinal plant that can regulate gastrointestinal function. Dendrobium huoshanense polysaccharides improved the motility and migration enhancement of GES-1 cells elicited by MNNG exposure (Xie et al., 2023). Dendrobium chrysotoxum Lindl is a commonly used drug in clinical treatment of chronic gastritis and precancerous lesions of gastric cancer, which has good clinical efficacy and medicinal value in inhibiting cell migration and invasion (Wang et al., 2021). Honokiol is a polyphenol isolated from the genus Magnolia with multiple biological functions. The results of Pan et al. (2013) suggested that Honokiol thwarts peritoneal dissemination and peritoneum or organ metastasis by mediating the expression of factors such as vimentin, Snail and Tpl2 to affect endoplasmic reticulum stress and suppress the process of EMT. The results of Liu et al. (2015) also suggested that Honokiol mediate Calpain-II/PPARγ/CRT pathway to suppresses peritoneal dissemination by activating ER stress and blocking EMT. Manpixiao Decoction is often used to improve the pathological progress of gastric mucosa in patients with precancerous lesions of gastric cancer. Manpixiao decoction is mainly used to improve the gastric mucosal pathology in patients with gastric precancerous lesions. These results of Li et al. (2022) indicated that Manpixiao decoction halted the precancerous lesion of gastric cancer progression via inhibiting EGFR/PI3K/AKT related EMT process. Hesperidin is a multifunctional citrus flavone, which is rich in many fruits and plants. It is reported that hesperidin reversed MNNG mediated upregulation of mesenchymal cell markers expression and downregulation of epithelial cell markers in gastric cancer rats by suppressing the PI3K/AKT pathway (Liang et al., 2022a). Eugenol is a phenolic component found in plants such as clove oil and cinnamon, which has been proven to have anticancer activity against various cancers. Palrasu et al. demonstrated that Eugenol inhibits invasion in rat models of gastric carcinogenesis induced by MNNG via regulating pro-invasive factors (Manikandan et al., 2010).

Cell migration and invasion play a key role in the occurrence and progress of gastric cancer. More and more studies have showed that phytochemicals inhibit cell migration and invasion of cells elicited by MNNG. Early intervention and long-term use of phytochemistry may have good clinical application prospects in MNNG induced gastric cancer metastasis and invasion.

### 3.3 Phytochemicals play an intervention and therapeutic role by influencing MNNG mediated apoptosis and autophagy

Apoptosis is a highly regulated process of cell death, while autophagy is a highly conserved self-defense mechanism (Xu et al., 2019; Lu et al., 2022). Regulating or interfering with cell apoptosis and autophagy may be one of the key mechanisms for inhibiting the occurrence and development of gastric cancer mediated by MNNG. Herein, we have summarized the regulation of phytochemicals on MNNG-mediated apoptosis and autophagy in this section (Table 2).

**TABLE 2:** Overview of the role of phytochemicals in cell apoptosis and autophagy.

Phytochemicals	Effects	Target	Subjects	References
Hesperidin	Induced cell autophagy	PI3K/AKT	Rats	Liang et al. (2022a)
Astragaloside IV	Inhibited cell autophagy	Ambra1, Beclin1, ATG5, LC3, p53 and p62	Rats	Cai et al. (2018)
Tagetes erecta flowers essential oils	Promoted cell apoptosis	Nrf2/HO-1 and NF-κB	Rats	Cui et al. (2021)
Weining granule	Promoted cell apoptosis	Bcl-2, caspase-3 and PTEN	Rats	Deng et al. (2019)
Xiao Tan He Wei Decoction	Promoted cell apoptosis	NF-kB	Rats and GES-1 cells	Xu et al. (2018)
EGCG	Promoted cell apoptosis	PI3K/Akt/mTOR	Rats	Zhu et al. (2021)
Corilagin	Promoted cell apoptosis	HMBG1/PI3K/AKT	Rats	Zhang et al. (2022)
Crocin	Promoted cell apoptosis	Nrf2/Hippo	Rats and GES-1 cells	Wu and Hui (2020)
Dendrobium huoshanense polysaccharides	Promoted cell apoptosis	Bcl-2, Bax, Caspase-3	GES-1 cells	Xie et al. (2023)
Crocetin	Promoted cell apoptosis	Bcl-2 and Bax	Gastric cancer cells	Bathaie et al. (2013a)
Erianin	Promoted cell apoptosis	HRAS/PI3K/AKT	precancerous lesions of gastric cancer cell model	Wang et al. (2021)
Ginsenoside Rg3	Promoted cell apoptosis	TIGAR	Gastric cancer cells	Lv et al. (2022)
MGN-3/Biobran	Promoted cell apoptosis	p53, Bax, Bcl-2 and caspase-3	Rats	Badr El-Din et al. (2016)
Eugenol	Promoted cell apoptosis	Bcl-2, Bcl-xL, Bax, Apaf-1, cytochrome C, caspase-9, caspase-3 and PARP	Rats	Manikandan et al. (2010)
Saffron aqueous extract	Promoted cell apoptosis	—	Rats	Bathaie et al. (2013b)
Myricetin	Promoted cell apoptosis	RSK2	Gastric cancer cells	Wang et al. (2014a)

It is reported that hesperidin reversed MNNG elicited gastric cancer through activating autophagy via the PI3K/AKT pathway (Liang et al., 2022a). Their results showed that hesperidin restore the expression of Beclin1, LC3-I/II and ATG5, which are reduced by long-term MNNG exposure. PI3K/AKT pathway plays an important role in this process. Astragaloside IV is a saponin extracted from Astragalus membranaceus, which is a traditional Chinese medicine widely used in the treatment of cancer. It provided a potential therapeutic strategy in regulating cell autophagy of gastric precancerous lesions and protecting the gastric mucosa in gastric precancerous lesions rats (Cai et al., 2018). It is suggested that possession of *Tagetes erecta* flowers essential oils has a protective effect on MNNG triggered gastric cancer by exerting an anti-apoptotic response through Nrf2/HO-1 and NF-κB pathways (Cui et al., 2021). It is reported that Weining granule could improve gastric cancer by promoting tumor cell apoptosis through regulating the expression of Bcl-2, caspase-3 and PTEN (Deng et al., 2019). The research founds that Xiao Tan He Wei Decoction could promoted apoptosis by increasing the expression of Bax/caspase-3 and decreasing the level of Bcl-2 (Xu et al., 2018). EGCG ameliorated pathological changes of gastric precancerous lesions and exerted proapoptotic effects on gastric precancerous lesions in rats through PI3K/Akt/mTOR pathway (Zhu et al., 2021). Corilagin is a natural ellagitannin with excellent anticancer pharmacological properties which has been proven to regulate the HMBG1/PI3K/AKT signal axis and trigger gastric cancer cell apoptosis induced by MNNG (Zhang et al., 2022). It was found that Crocin suppressed the Nrf2/Hippo pathway and then increased cell apoptosis mediated by MNNG (Wu and Hui, 2020). Dendrobium huoshanense polysaccharides promoted the MNNG elicited cell apoptosis by regulating the levels of apoptosis Bcl-2, Bax, Caspase-3 (Deng et al., 2019). Crocetin is a carotenoid derived from saffron, which increased MNNG induced cell apoptosis of gastric cancer cells by regulating the expression of Bcl-2 and Bax (Bathaie et al., 2013a). Verification through experiments found that Erianin, the main active ingredients of Dendrobium officinale, could significantly induce cell apoptosis triggered by MNNNG in a dose-dependent manner through the HRAS-PI3K-AKT pathway (Wang et al., 2021). Lv et al. (2022) found that Ginsenoside Rg3 could suppress TIGAR and induce cell apoptosis in MNNG induced gastric precancerous lesion rats. Nariman et al. reported that MGN-3/biological bran exerts chemopreventive effects on gastric carcinogenesis in rats by regulating gastric cancer cell apoptosis (Badr El-Din et al., 2016). Eugenol, which can manipulate the balance between proapoptotic and anti-apoptotic proteins, is an attractive candidate for preventing MNNG induced gastric cancer progression (Manikandan et al., 2010). Saffron and its components have excellent anticancer activity. Saffron water extract inhibits MNNG-triggered gastric cancer in stomach of rats by promoting cell apoptosis (Bathaie et al., 2013b). Actinidia valvata Dunn extract exhibits the preventive effect on MNNG-induced gastric carcinogenesis through the regulation of cell apoptosis by regulating the expression of Bcl-2 (Wang et al., 2014a).

In conclusion, the above studies showed that phytochemicals may be used as promising candidates against MNNG-induced gastric cancer by mediating cell apoptosis and autophagy.

### 3.4 Regulation of inflammation and oxidative stress mediated by MNNG

Reactive oxygen species mediated cell and tissue damage is a common pathway in the occurrence and development of many diseases. Almost all tumor cells have oxidative stress imbalances, and continuous oxidative stress plays a crucial role in many tumors such as gastric cancer. Inflammation, as one of the top ten characteristics of tumors, also plays a very important role in MNNG induced gastric cancer. We summarized the effects of phytochemicals on oxidative stress and inflammation to interfere with the occurrence and development of MNNG-induced gastric cancer, in order to provide theoretical basis and strategies for subsequent research and clinical application (Table 3).

**TABLE 3:** Overview of the effect of phytochemicals on inflammation and oxidative stress mediated by MNNG.

Phytochemicals	Effects	Target	Subjects	References
Carvacrol	Antioxidant and anti-inflammatory	—	Rats	Gunes-Bayir et al. (2022)
Tagetes erecta flowers essential oils	Antioxidant and anti-inflammatory	Nrf2/HO-1/NF-κB	Rats	Cui et al. (2021)
Scutellarin	Antioxidant and anti-inflammatory	—	Gastric cancer cells and Rats	Sun and Meng (2022)
Sargassum pallidum aqueous extract	Antioxidant and anti-inflammatory	anti-inflammatory and pro-inflammatory cytokines	Paclitaxel	Zhang et al. (2012)
Dendrobium officinale polysaccharides	Antioxidant	NRF2, HO-1 and NQO-1	Rats	Zhao et al. (2019a)
Dendrobium officinale Polysaccharides	Antioxidant	Wnt/β-Catenin	Rats	Zhao et al. (2019b)
Crocetin	Antioxidant	—	Rats	Bathaie et al. (2013a)
Lycopene	Antioxidant	SOD, CAT, and GPx	Rats	Luo and Wu (2011)
Purslane polysaccharides	Antioxidant	SOD, CAT, GSH-Px	Rats	Li et al. (2014)
Weipiling decoction	Anti-inflammatory	NF- κB	Mice	Yang et al. (2022)
Manpixiao decoction	Anti-inflammatory	EGFR/PI3K/AKT	Rats	Li et al. (2022)
SHPPB2	Anti-inflammatory	anti-inflammatory and pro-inflammatory cytokines	Rats	Han et al. (2018)

Carvacrol is a dietary polyphenol derived from plants that has been proven to have a wide range of biological activities for human health. These results of Gunes-Bayir et al. (2022) indicated that Carcinol exhibited significant antioxidant and anti-inflammatory effects on MNNG induced gastric carcinogenesis in rats. The findings of Ayse et al. indicated that *T. erecta* flowers essential oils has a protective effect on MNNG induced gastric cancer by exerting antioxidative stress and anti-inflammatory response via Nrf2/HO-1 and NF-κB pathways (Cui et al., 2021). Scutellarin is a kind of bioactive flavonoid obtained from plants such as Erigeron breviscapus. Sun and Meng (2022) reported that Scutellarin exhibited antioxidant and anti-inflammatory effects on MNNG-triggered gastric carcinogenesis in rats. *Sargassum pallidum* aqueous extract has been found to be against MNNG-mediated inflammation and oxidative injury by enhancing anti-inflammatory cytokines, decreasing pro-inflammatory cytokines, maintaining normal antioxidant enzyme activity by inhibiting lipid peroxidation in gastric mucosa (Zhang et al., 2012). *Dendrobium officinale* polysaccharides protect against MNNG-induced precancerous lesions of gastric cancer through activating NRF2, HO-1 and NQO-1 (Zhao et al., 2019b). *Dendrobium officinale* Polysaccharides regulate Wnt/β-catenin pathway, which played an antioxidant role in inhibiting MNGG induced precancerous lesions in rats (Zhao et al., 2019b). The study of Bathaie et al. (2013a) demonstrated the antioxidant activity of Crocetin against gastric cancer mediated by MNNG that may benefit gastric cancer treatment. Purslane polysaccharides are the main bioactive components of *Portulaca oleracea* and have extensive pharmacological effects. Li et al. (2014) indicated the interventional effects of Purslane polysaccharides on the oxidative damage in MNNG-elicited gastric cancer rats by regulating inflammatory and antioxidant factors. Lycopene is a kind of carotenoid and a powerful antioxidant, which mainly exists in tomatoes and tomato products. Lycopene increased MDA concentration and enhanced antioxidant enzyme activities such as SOD, Catalase and Glutathione peroxidase in MNNG-induced gastric cancer rats to exert its antioxidant effect in gastric cancer (Luo and Wu, 2011). Weipiling decoction attenuated MNNG-induced gastric precancerous lesions, including epithelial shedding, intestinal metaplasia, cavity fusion, INF-γ production, dysplasia, pro-inflammatory Th1-cell infiltration, and basement membranes with asymmetrical thickness, pointing towards that Weipiling decoction prevents inflammation in the gastric mucosa (Yang et al., 2022). SHPPB2 was a complicated sulfated fucoidan purified from Sargassum henslowian. SHPPB2 could significantly promote the proliferation of spleen cells in gastric cancer rats induced by ConA or LPS, improve anti-inflammatory cytokines secretion, and reduce pro-inflammatory cytokines (Han et al., 2018). The research results of Li et al. (2022) indicated that Manpixiao decoction reduces inflammatory cytokines such as IL-1α, IL-7, CSF-1 and CSF-3 in serum of precancerous lesion of gastric cancer rats induced by MNNG.

The above studies demonstrated that phytochemicals could interfere with the occurrence and development of gastric cancer induced by MNNG through influencing oxidative stress and inflammation, providing a new strategy for early intervention in gastric cancer.

### 3.5 Inhibition of MNNG induced angiogenesis

Many studies have found that angiogenesis plays a crucial role in the occurrence and development of cancers such as gastric cancer (Da et al., 2015; Huang et al., 2017; Zang et al., 2017; Da et al., 2019). Accumulating evidence demonstrated that phytochemicals prevent MNNG induced gastric cancer by inhibiting angiogenesis (Wen et al., 2021; Gao et al., 2022; Zeng et al., 2022). In this section, we summarized the phytochemicals that suppress MNNG-induced angiogenesis and analyzed their mechanisms.

Dehydroevodiamine is a quinazoline alkaloid isolated from Euodiae Fructus. It was reported that Dehydroevodiamine has an intervention effect on MNNG-induced angiogenesis and inhibit the expression of HIF-1α/VEGF pathway (Wen et al., 2021). Weining granule could improve gastric cancer by suppressing MNNG-induced angiogenesis (Deng et al., 2019). Ginsenoside Rg3 is an active saponin extracted from ginseng. Ginsenoside Rg3 has the function to inhibit angiogenesis and regulate microvascular abnormalities in gastric precancerous lesions rats induced by MNNG via regulating the levels of Glut1 and Glut4 (Zeng et al., 2022). Palrasu et al. reported that Eugenol could inhibit MNNG induced angiogenesis in gastric carcinogenesis by regulating numerous angiogenic factors (Manikandan et al., 2010). Results of Zeng et al. (2018) indicated that Weibixiao could alleviate early gastric angiogenesis induced by MNNG and alleviate microvascular abnormalities in gastric precancerous lesions rats through the ERK1/Cylin D1 pathway and angiogenic factors. Atractylolide III is the main active ingredient in the rhizome of Atractylodes macrocephala and exhibits anti-tumor activity in various tumors such as gastric cancer. It is reported that Atractylenolide III attenuated angiogenesis by suppressing the expression of HIF-1 α and VEGF-A, while reducing microvascular abnormalities and early angiogenesis in gastric precancerous lesions rats modles through regulation of delta-like ligand 4 (Gao et al., 2022).

### 3.6 Regulation of metabolism and microbiota in MMNG induced gastric cancer

As an important organ with abundant flora and concentrated metabolism, the stomach may be more affected by flora and metabolism. With the rise of metabolomics and microflora, more and more studies have confirmed that metabolism and microbial communities play an important role in the occurrence and development of MNNG induced gastric cancer. In this section, we conclude and summarize the intervention effects of phytochemicals on the occurrence and development of MNNG induced gastric cancer by regulating metabolism and flora.

Glycolysis is considered one of the hallmarks of gastric cancer and other cancers. The findings of Cai et al. (2019) implied that abnormal glycolysis induced by MNNG in gastric precancerous lesions in rats was relieved by Weipixiao decoction. Peng et al. (2022) found that Xiaopi granules ameliorated MNNG mediates gastric epithelial dysplasia by intervening in metabolism, digestion, coagulation, and other related physiological/pathological processes. Dendrobium huoshanense polysaccharides might protect against MNNG-induced injury of gastric mucosa cells mediated by nicotinamide and energy metabolism related pathways (Xie et al., 2023). They found that Dendrobium huoshanense polysaccharides increased famotidine, 1-methylnicotinamide, acetyl-L-carnitine, N4-acetylsulfamethoxazole, choline metabolites, and significantly reduced valproic acid, L-cystine, propoxy, oleic acid and 6-O-demethyldonepezil, thus regulating the metabolic process of gastric cancer cells. Compared with the MNNG group, scutellarin supplementation revealed an upregulation in the rats’ body weight. In addition, significant changes in DNA density, mucus content, LDH content, and acidity induced by scutellarin were also observed (Sun and Meng, 2022). Crocetin and saffron aqueous extract reversed the changes of some biochemical parameters in gastric cancer tissues induced by MNNG in rats, thereby exerting an anticancer effect (Bathaie et al., 2013a; Bathaie et al., 2013b).

Apigenin is one of the most common flavonoids, which is abundant in parsley, celery, passion fruit, chamomile, and other vegetables and fruits. Apigenin could significantly reduce the colonization of *H. pylori* in Mongolian gerbils with atrophic gastritis and gastric cancer, as well as the infiltration of neutrophils and monocytes induced by *H. pylori* and MNNG (Kuo et al., 2014). Hua Zhuo Jie Du is a herbal prescription, which is frequently used to treat chronic atrophic gastritis. Huazhuo Jiedu protected chronic atrophic gastritis induced by MNNG by regulating the microbiota and its metabolites (Zhou et al., 2021).

Exploring the effects and mechanisms of phytochemicals on gastric metabolism and flora provide some new strategies for the intervention and the treatment of MNNG related gastric cancer. In the future, it may become one of the research hotspots for early intervention and treatment of gastric cancer.

### 3.7 Regulation of immunity in MMNG induced gastric cancer

Excellent immunity plays a very powerful role in healing wounds, resisting pathogens, repairing tissues, and inhibiting the occurrence and progression of many cancers. Cancer cells such as gastric cancer cells evade immune detection and elimination through various mechanisms, thereby promoting cancer occurrence and progression. A strong immune system is crucial for maintaining the health and quality of life of cancer patients such as gastric cancer. Therefore, it is crucial to study the regulatory effects of phytochemicals on immune function.

Li et al. (2014) summarized that enhancing immune response may be the reason for the anticancer effect of Purslane polysaccharides in MNNG elicited gastric cancer. Their experiment found that Purslane polysaccharides not only increased the peripheral blood leukocyte count, thymus and spleen indices of gastric cancer rats, but also significantly promoted spleen cell proliferation and enhanced serum cytokine production. Administration of lycopene to MNNG induced gastric carcinoma rats significantly upregulated immunity activities by increasing the levels of IgG, IgA, and IgM, as well as IL-2, IL-4, IL-10, while reducing IL-6 levels, thereby reducing the risk of gastric cancer (Luo and Wu, 2011). The results of Han et al. suggested that SHPPB2 (a sulfated fucoidan) could improve immune function via increasing the body weight, improving immune organ index, promoting spleen cell proliferation, and improving the secretion of anti-inflammatory cytokines, but reducing pro-inflammatory cytokines of gastric cancer rats mediated by MNNG (Han et al., 2018). SMPA is a polysaccharide component extracted from Salvia miltiorrhiza. Studies of various immunological activities revealed that SMPA can significantly stimulate the proliferation of splenocytes cells, enhance the killing activity of NK cells and cytotoxic T lymphocytes, and promote the phagocytic function of macrophages in gastric cancer rats induced by MNNG (Wang et al., 2014b). It is reported that *Sargassum pallidum* aqueous extract enhanced the immunological function by improving the secretion of anti-inflammatory cytokines, but reducing pro-inflammatory cytokines in MNNG triggered gastric cancer rats (Zhang et al., 2012).

The immune capacity of the body plays a key role in the occurrence and development of various cancers, including MNNG related gastric cancer, and many tumor cells promote tumor progression through immune escape. Therefore, studying the role of phytochemicals in regulating the immune system of the body has great application prospects.

### 3.8 Other relevant mechanisms

In addition to the above modes of mechanisms, phytochemicals also play a preventive and treatment role in gastric cancer mediated by MNNG through other relevant mechanisms.

Sancao Tiaowei decoction has been used for the prevention and treatment of precancerous lesions in gastric cancer. These results suggested that Sancao Tiaowei decoction could significantly inhibit the pathological progress of gastric mucosa in rats with precancerous lesions of gastric carcinoma induced by MNNG (Cai et al., 2022). Ginsenoside Rb1 could prevent the occurrence and progression of gastric precancerous lesions mediated by MNNG, which might be due to reduced protein expression, nuclear translocation of β-catenin and interfering with β-catenin/TCF4 interaction (Zeng et al., 2021). Elian granules might play a critical role in the treatment of MNNG induced precancerous lesions of gastric cancer by maintaining the integrity of the gastric mucosa epithelium, the orderly arrangement of glands and inhibiting the infiltration of inflammatory cells through MAPK pathway (Yi et al., 2022). The results of Pan et al. (2013) suggested that Magnolol inhibits the growth of gastric cancer and peritoneal dissemination by inducing endoplasmic reticulum response to radicalization. Calycosin passes through integrin β1/NF-κB/DARPP-32 pathway to protect gastric mucosa from damage in MNNG induced precancerous lesions of gastric cancer (Li et al., 2020). It is demonstrated that Granule Dendrobii was are effective in treating chronic atrophic gastritis caused by MNNG exposure via reversing gastric atrophy and intestinal metaplasia, improving the histopathology of gastric mucosa (Wu et al., 2022). Study of Zhang et al. (2018) has reported that astragaloside IV alleviated MNNG induced abnormal glycolysis of the precancerous lesions of gastric carcinoma. Weiqi decoction attenuated gastric mucosa blood flow disorder and HIF-1 α pathway alleviates MNNG mediated chronic atrophic gastritis with precancerous lesions (Yin et al., 2019).

## 4 Discussion

Phytochemistry substances are bioactive compounds found in vegetables, fruits, nuts, tea, fruits and herbal medicine, which are beneficial to human health. They have been proven to have extensive anticancer activities against MNNG induced gastric cancer by regulating a variety of physiological and pathological processes, such as inhibiting cell proliferation, migration and invasion, blocking angiogenesis, regulating metabolism and microbiota, enhancing immunity, regulating apoptosis and autophagy.

Gastric cancer patients usually cannot be completely cured through conventional chemoprevention strategies, therefore, preventing, suppressing, and/or delaying the onset of these cancers is crucial. The commonly used chemotherapy drugs also have strong toxic and adverse effects on normal cells in the human body and are prone to drug resistance. In addition, long-term use can limit efficacy and reduce quality of life. Development of therapeutic remedies with less side effects and lower chemoresistance is required. Natural phytochemicals are emerging as alternative resources to combat gastric carcinoma. Compared with traditional cancer treatment drugs such as gastric cancer, phytochemicals have many advantages, such as wide source, easy application and excellent anti-cancer effect. In addition, it is critical that phytochemicals have high safety, most of which have few side effects, and even some phytochemicals have no toxic effects on normal cells. In addition, many phytochemicals can trigger a variety of anti-cancer mechanisms, such as anti-proliferation, anti-migration, promoting apoptosis, regulating immunity, and so on. They have synergistic effects in many ways, have multiple beneficial effects on health and gastric cancer patients and more targets than some common chemical anti-cancer drugs. Studies have found that phytochemicals can enhance the efficacy of chemotherapy drugs, reduce the side effects of chemotherapy drugs, and reduce the incidence of drug resistance. However, some phytochemicals with excellent anti-cancer effect have problems such as low bioavailability, difficulty in measuring blood concentration and lack of standardization.

The application of phytochemicals in the clinical treatment and prevention of gastric cancer is still greatly limited. Therefore, it is particularly important to better apply phytochemistry to the clinical early intervention and treatment of MNNG mediated cancer. We need to better clarify the effects and mechanisms of phytochemicals in the intervention and treatment of MNNG related gastric cancer, laying a foundation for clinical application. It is urgent to establish methods that can accurately determine the concentration of phytochemistry in blood or target tissues. More clinical trials are needed, especially in combination with standardized treatment regimens currently in use. There is also a need to find better forms of drug delivery to improve the bioavailability of phytochemicals. We can deliver phytochemicals through the exocrine pathway in research to improve the bioavailability and targeting of phytochemicals in the intervention and treatment of MNNG induced gastric cancer. In addition, the exosomes of phytochemistry can also be directly extracted and acted on MNNG triggered malignant transformed cells and gastric cancer cells to observe whether they can enhance the anti-cancer effect and bioavailability.
